# Bonobos and chimpanzees overlap in sexual behaviour patterns during social tension

**DOI:** 10.1098/rsos.242031

**Published:** 2025-03-05

**Authors:** Jake S. Brooker, Christine E. Webb, Edwin J. C. van Leeuwen, Stephanie Kordon, Frans B. M. de Waal, Zanna Clay

**Affiliations:** ^1^ Department of Psychology, Durham University, South Road, Durham DH1 3LE, UK; ^2^ Department of Human Evolutionary Biology, Harvard University, 11 Divinity Avenue, Cambridge, MA 02138, USA; ^3^ Animal Behaviour and Cognition, Department of Biology, Utrecht University, Padualaan 8, Utrecht 3584 CA, The Netherlands; ^4^ Department for Comparative Cultural Psychology, Max Planck Institute for Evolutionary Anthropology, Deutscher Platz 6, Leipzig 04103, Germany; ^5^ Department of Psychology, Emory University, 36 Eagle Row, Atlanta, GA 30322, USA

**Keywords:** bonobo, chimpanzee, reassurance, sexual contact, social tension, species variability

## Abstract

Sexual behaviour during tense social situations is extensively documented in various animals. Bonobos, our closest living relatives alongside chimpanzees, habitually perform genital contacts during social tension, which is thought to enhance cooperation and conflict management. While chimpanzees also engage in genital contacts during these contexts, the two sister species have yet to be compared systematically, which may have led to inaccurate assumptions. To address this, we directly compared genital and non-genital affiliation among sanctuary-living bonobos and chimpanzees during two socially tense contexts—post-conflict and pre-feeding. Following conflicts, we observed triadic affiliation between bystander–victim pairs and reconciliation between aggressor–victim pairs. Additionally, we experimentally induced a pre-feeding context to examine affiliative contact between group members. During post-conflict contexts, bonobos used genital contacts more than chimpanzees. However, both species used genital contacts comparably during pre-feeding affiliation, although female bonobos and male chimpanzees were most likely to initiate them. In addition, we found group-level variation indicating an influence of demographic factors. Our results indicate that chimpanzees and bonobos overlap significantly in their use of genital contacts during periods of social tension. Given similar evidence in humans, our results support the notion that this was a trait probably also present in our last common ancestor.

## 1. Introduction

Non-conceptive sexual behaviour (henceforth: sociosexual behaviour) is commonplace in human society, extending beyond reproduction to serve a variety of social functions [1–3]. Across cultures, sociosexual behaviours play a significant role in fostering trust and reinforcing social bonds [1–4]. Sociosexual behaviours have also been linked to psychological and emotional well-being, with studies demonstrating their positive impact on reducing stress and enhancing interpersonal connection [1,2,5,6]. Sociosexuality also appears to be widespread throughout the animal kingdom [7,8], particularly among non-human primates (henceforth ‘primates’; see [9] for a review). Exploring sociosexuality in primates provides a valuable comparative framework for understanding the evolutionary origins of these behaviours, offering insights into their broader significance across species, including our own.

Among the primates, bonobos (*Pan paniscus*) are most known for their rich and heightened sexuality [10–13] where sexual behaviour plays a key role in both female and male bonobo social development, emerging already during the first year of life [14]. Initially, most bonobo sexual interactions begin between mother–infant pairings when either party is distressed or anxious [15,16]. As they mature, bonobos engage in sexual behaviour across a variety of contexts with their peers, including play [17], but notably during periods of social tension, such as during conflict management and resolution of social tension and feeding [12,15,18–20].

Genito-genital (GG) rubbing—when two individuals, typically females, embrace ventro-ventrally and swing their hips laterally with their vulvae or penises in contact [12,21]—is a species-typical behaviour in bonobos thought to primarily function to enhance social bonding and cooperation [20], having been linked with food sharing and closeness during feeding [22]. These findings and observations of GG rubbing during intergroup encounters [10,23] and within-group fusions [24] indicate that this sexual behaviour may also facilitate social tolerance. While adult female bonobos are known for their heightened sexual relationships, males also use various forms of sociosexual behaviour, including GG rubbing and mounting, with other males [25,26] and females [27]. In bonobos, male–male mounting often occurs during socially tense contexts where it may also reflect dominance relationships, whereby higher rankers tend to mount lower rankers, although not always [25,28]. In addition, immature female and male bonobos engage in homosexual and heterosexual sociosexual contacts [27,29].

Although much more attention has been placed on bonobo sexuality, there is evidence that chimpanzees also engage in sociosexual behaviour in similar contexts. Mixed-sex and same-sex genital touching and mounting have been observed across ages in chimpanzees [30] and, like bonobos, occur frequently during chimpanzee play [31]. Genital touching—hand or foot contact to another’s anogenital region—is used often in greetings [32], where it appears to represent a form of reassurance, occurring often during post-conflict (PC) affiliation with uninvolved bystanders and reconciliation with aggressors too [33–35]. Similarly in chimpanzees, GG contact or rump-to-rump contact—where individuals face away from one another and place their rumps (males) or swellings (females) in contact, akin to bonobo GG rubbing—occurs often during periods of social tension [30] and may have a reassuring function [36]. Although much more habitual and ubiquitous in bonobos, same-sex GG rubbing has been observed in a captive group of female chimpanzees and was associated with grooming relationships, suggesting an association with social bonding [37]. In addition, oral–genital contact has been observed in captive chimpanzees during contexts such as play [31] and social tension relief [38]. Sexuality therefore appears to play a role in both bonobo and chimpanzee social life, and different forms may flexibly emerge according to context and population.

In bonobos, sexual behaviour has predominantly been studied regarding its tension-relieving and social-bonding function [12,18,19,39]. GG rubbing is common during periods of within-group tension, such as PC periods [40,41] and during competitive situations like feeding [12,42]. Similarly, chimpanzees also often mount and touch genitals, including male–male testicle shaking, following conflicts and during intergroup and predator encounters [33,35,43,44]. Sandel & Reddy [30] found that genital contacts in wild chimpanzees were most common during socially tense contexts, including subgroup fusions and territorial behaviour. While individuals of all ages and sexes were seen to mount and touch genitals, most sociosexual patterns were seen among adult males. Wild chimpanzees have also been reported to hold the genitals of their social partners during intergroup encounters [45].

Although rarer among the other great apes, wild gorillas and wild orangutans have sometimes also been observed to use sexual behaviour in homosexual pairings [46,47]. Like *Pan,* male homosexual interactions in wild orangutans have been associated with social tension and affiliation [46]. These observations across great apes coupled with similar apparent functions of sociosexual behaviour in humans [1,2,5,6,48] indicate that affiliative genital contact may have been a trait already present in our last common ancestor of extant hominids, which may, among other possible functions, help to manage social relationships and periods of social tension [2,12,30].

Various forms of reassuring contact behaviours have been widely documented in both captive and semi-wild groups of bonobos and chimpanzees to function as consolation (i.e. stress-relieving friendly contact offered by non-involved third-parties), reconciliation (i.e. affiliative contact between recent conflict opponents to repair social bonds) and as reassurance broadly to prevent tension from escalating [18,40,49–52]. Within these contexts, both species appear to use sociosexual behaviours between various age and sex combinations [18,19,30,34,53,54]. Thus, genital contacts may play a vital role in maintaining collective harmony, mitigating aggression and restoring social stability following tension escalation.

However, despite its apparent association with tension management in both species, patterns in their use of sexual behaviour have not been extensively compared. A comparative approach can help to elucidate the evolutionary foundations for sociosexual behaviour, what functions they may serve and the contexts they are common in. As humans also use sociosexual behaviours to navigate relationships and mitigate social tension, exploring these contexts in our two closest relatives may elucidate species-specific strategies and shared ancestral traits that may have enabled early hominins to balance competition and cohesion in resource-limited environments.

To address this, we systematically compared the tendencies of large populations of sanctuary-living bonobos and chimpanzees to engage in genital contact behaviour when navigating social tension contexts. To do so, we compared the tendency for genital contact to occur during affiliative interactions in two specific contexts: PC (*part 1*) and pre-feeding (*part 2*). Within PC contexts, we compared bonobos and chimpanzees in their tendency to use genital contact between distressed victims in triadic contact interactions with uninvolved bystanders (*part 1A*) and during reconciliation with their previous aggressors (*part 1B*). As sexual behaviours are thought to be particularly significant to managing bonobo social life [10,12,18], we predicted that for PC affiliation and pre-feeding affiliation contact among bonobos would be more likely to feature genital contact as compared with chimpanzees. In addition, given that genital contacts occur in all age and sex combinations in the *Pan* apes, we tested for within-species trends by comparing different ages and sexes in their tendency to use and receive genital contact in these contexts. We predicted that, as genital contacts may contribute to strengthening social ties [18,30], genital contacts would be used more by older individuals of both species and more between female–female pairs in bonobos and male–male pairs in chimpanzees.

## 2. Methods

### 2.1. Study sites and subjects

We collected data at two African great ape sanctuaries: Lola ya Bonobo Sanctuary (hereafter: Lola ya Bonobo) in the Democratic Republic of the Congo and Chimfunshi Wildlife Orphanage Trust (hereafter: Chimfunshi) in the Copperbelt Province of Zambia. We conducted observations of bonobos at Lola ya Bonobo during July–September 2019 (logging 600 h of observation) and observations of chimpanzees at Chimfunshi during March–August 2019 (logging 800 h of observation). Lola ya Bonobo houses three groups of bonobos in enclosures ranging from 15 to 20 hectares with rainforest, lake, swamp, stream and open grass areas. Chimfunshi houses four groups of chimpanzees in enclosures ranging from 47 to 190 acres with miombo woodland [55,56]. At Lola ya Bonobo, bonobos sleep at night together in dormitories. At Chimfunshi, chimpanzees nest outside unless kept indoors for monitoring or medical intervention. Both sanctuaries house wild-born individuals, orphaned and rescued from the pet and bushmeat trades, as well as sanctuary-born individuals. In both sanctuary environments, the apes can roam and forage independently, while supported by an on-site caregiving team who provision them at least twice per day with a variety of fruits and vegetables.

We observed *n* = 54 bonobos across *n* = 3 groups (*n*: B1 = 22; B2 = 18; B3 = 14) at Lola ya Bonobo and *n* = 75 chimpanzees across *n* = 2 groups (*n*: C1 = 25; C2 = 50) at Chimfunshi. For the comparison of sexual behaviour during naturally occurring PC periods (*part 1*), we compared two groups of bonobos (B1 and B2) and one group of chimpanzees (C2), due to limited observational time. For the experimental comparison of sexual behaviour during pre-feeding affiliation (*part 2*), we compared all five groups. We excluded one individual from analyses in *part 1* and five individuals from *part 2* due to complete absence during respective observations. Age and sex composition of all groups is provided in table 1.

**Table 1.** Social compositions of bonobo and chimpanzee groups from Lola ya Bonobo Sanctuary and Chimfunshi Wildlife Orphanage Trust, respectively. Chimpanzees at Chimfunshi include a mix of subspecies. *Part 1* reflects all individuals eligible for PC analyses, and *part 2* reflects all individuals present during at least one pre-feeding session.

Part 1: Genital contact during PC affiliation.					
species	group	total	infants^a^	juveniles^a^	adults^a^
			F|M^b^	F|M^b^	F|M^b^
bonobo (*Pan paniscus*)	1 (B1)	22	0|1	5|5	7|4
	2 (B2)	17^c^	0|0	7|4	1|5
chimpanzee (*Pan troglodytes*)	2 (C2)	50	4|2	6|7	23|8
total	3	89	4 | 3	18 | 16	31 | 17

Part 2: Genital contact during pre-feeding affiliation.					
species	group	total	infants^a^	juveniles^a^	adults^a^
			F|M^b^	F/M^b^	F/M^b^
bonobo (*Pan paniscus*)	1 (B1)	22	0|1	5|5	7|4
	2 (B2)	16^d^	0|0	7|4	1|4
	3 (B3)	14	0|0	4|2	4|4
chimpanzee (*Pan troglodytes*)	1 (C1)	25	0|2	5|1	11|6
	2 (C2)	47^d^	4|1	5|7	23|7
total	5	124	4|4	26|19	46|25

^a^ Age in years: infants = 0−2; juveniles = 3−11; adults = 12+.

^b^ F = number of females; M = number of males.

^c^ Total excludes Kodoro due to lack of observations.

^d^ Total excludes Eleke (B2), Kodoro (B2), Masya (C2), Mikey (C2) and Mumba (C2) due to absence from all experimental sessions.

### 2.2. Data collection and coding

We compared the use of genital contact behaviours during two affiliative contact interactions in two socially tense contexts: PC (*part 1*) and pre-feeding (*part 2*). For both parts, contact-affiliation behaviours were categorized as either ‘genital contact (GC)’ (including genital touch, GG contact and mount) or ‘non-GC’ (including body kiss, contact sit, embrace, finger/hand in mouth, grasp hand, groom, hunch-over, mount walk, mouth kiss, pat, peer, play and touch). We applied the same ethogram to behavioural coding for both contexts in both species (see electronic supplementary material, SI.1.1). All code and data are accessible via an OSF folder [57].

#### 2.2.1. Part 1: Post-conflict observations

We systematically collected PC observations at both sanctuary sites to gather comparative data on the use of triadic victim contacts and reconciliation contacts in bonobos and chimpanzees. We all-occurrence sampled [58] instances of dyadic and polyadic aggressions (see electronic supplementary material, SI.1.2 for an extensive ethogram) and conducted 5 min focal-follows of victims who expressed victim signalling behaviour (e.g. whimpering, screaming and bared-teeth displays) following the encounter. For *part 1A*, we also followed individuals who spontaneously became distressed, exhibiting similar victim signalling behaviour without conflict occurring. Including tantrums by infants and juveniles, these periods are known as post-distress (PD) periods. Previously, PC/PD events compared with matched controls (MC) have demonstrated that both bonobos and chimpanzees use various forms of PC affiliation, including triadic contact (defined as contact between a distressed individual and a non-involved third-party [59]), consolation (defined as stress-relieving contact solicited from an uninvolved third-party towards a distressed conspecific [60]) and reconciliation (defined below [34]). As we aimed to compare individuals in their forms of PC behaviour broadly, we decided to forgo sampling MCs and focus on collecting sufficient PC and PD events.

We conducted behavioural coding of all social interactions that victims engaged in during PC and PD events using the video software ELAN [61,62]. For *part 1A*, triadic victim contact was defined as an affiliative interaction between the victim and an uninvolved bystander that included at least one contact behaviour [63]. We collected *n* = 194 PC/PD events across both species that featured at least one instance of triadic victim contact (B1 = 48; B2 = 31; C2 = 115). For *part 1B*, reconciliation contact was defined as an affiliative interaction between the victim and aggressor that included at least one contact behaviour [63]. We collected *n* = 44 PC events across both species that featured at least one instance of reconciliation (B1 = 4; B2 = 9; C2 = 31). To assess inter-coder reliability (ICR; J.S.B. and S.K. double-coded 20.2% of bonobos events (*n* = 16) and two independent coders coded 28.7% of chimpanzee sessions (*n* = 33). Results for reliability of coded behaviour indicated strong agreement (bonobo *κ* > 0.85; chimpanzee *κ* > 0.85), and individual identities were near perfect (bonobo *κ* > 0.90; chimpanzee *κ* > 0.95).

#### 2.2.2. Part 2: Pre-feeding affiliation observations

We adapted an established experimental measure of co-feeding tolerance—the *peanut swing* [64]—to create a controlled arena of social tension to systematically compare pre-feeding affiliation behaviour in sanctuary-living apes. This experiment features a trough-like receptacle, constructed from a bamboo pole, measured to 1 m in length per five individuals and filled with 12 peanuts per eligible individual (i.e. 3 years old and older) in the group. The trough is then swung at chain-link enclosures, thereby systematically distributing a valuable, yet depletable, resource in a limited spatial zone. Once the peanuts have landed inside the enclosure, researchers count the total crowd size gathered within 1 m of the fallen peanuts, in what is termed the co-feeding zone, at 15 s intervals up to 2 min—typically by which time most of the peanuts have been eaten. By averaging these values and comparing across groups, one can assess the relative social tolerance of *Pan* groups, whereby groups with higher group proportions gathering in the co-feeding zone are interpreted as being more socially tolerant [65]. In our deployment of the *peanut swing*, we introduced a 5 min pre-feeding period. We initiated this period by announcing an upcoming feeding to the apes using calls and sounds familiar to them and recorded all affiliative interactions during this pre-feeding period. We used two stationary cameras per trial to cover as much of the enclosures as possible, with additional handheld cameras to help with identification of individuals in larger groups.

We conducted *n* = 60 sessions across all five groups. However, to best reflect typical group dynamics, we only analysed data from sessions where at least 80% of the group was seen prior to the feeding. This resulted in a total session *n* of B1 = 8; B2 = 10; B3 = 10; C1 = 9; C2 = 8. Across these sessions, *n* = 124 apes (B1 = 18−22; B2 = 14−16; B3 = 12−14; C1 = 19−25; C2 = 41−47) were present for at least one session. We defined a pre-feeding affiliative interaction as at least one instance of contact affiliation between two individuals during the 5 min pre-feeding window. We coded all pre-feeding interactions using ELAN [61], including behaviour, behaviour type and initiator. We coded initiators as the individuals within the dyad who performed an approach or addressed a communicative signal to the other prior to the specific contact in question. The coding scheme applied the same contact-affiliation ethogram from *part 1* for pre-feeding interactions (see electronic supplementary material, SI). Due to the nature in which we filmed sessions, it was not clear to determine skin-to-skin contact during peering bouts, and so we proceeded without this behaviour type for pre-feeding affiliation. To assess ICR, we coded 8% of bonobo sessions (*n* = 3) and 13% of chimpanzee sessions (*n* = 3) twice. Results for reliability of coded behaviour indicated strong agreement (bonobo *κ* > 0.90; chimpanzee *κ* > 0.80), and individual identities were also at least strong (bonobo *κ* > 0.80; chimpanzee *κ* > 0.90). In addition, reliability of coding the same initiator for each behaviour was also strong (bonobo *κ* > 0.80; chimpanzee *κ* > 0.80).

## 3. Part 1: Variability in *Pan* post-conflict genital contact

### 3.1. Part 1A: Triadic contacts

#### 3.1.1. Analysis

To test if bonobos are more likely than chimpanzees to use genital contact during triadic victim interactions, we fitted a generalized linear mixed model (GLMM) in RStudio (v. 1.3.1093 [66]) using the function glmer of the package lme4 (v. 1.1-28 [67]). Specifically, we analysed a behaviour-level data frame of *n* = 784 observation rows, where one row constituted one contact behaviour that occurred during *n* = 194 PC/PD events (bonobo PC/PD *n* = 79; chimpanzee PC/PD *n* = 115). We fitted two GLMMs with binomial error distributions (testing species differences: *model 1.1.1*; testing group differences: *model 1.1.2*) in which the response variable was a dichotomous 1/0 variable (1 = behaviour involved genital contact; 0 = behaviour did not involve genital contact).

For *model 1.1.1*, the fixed-effects structure included species, bystander age, bystander sex, victim age, victim sex and maternal kinship (hereafter: kinship) of the bystander–victim pairing. We defined each dyad as ‘kin’ or ‘non-kin’ depending on whether they shared a maternal genetic relationship or not, respectively. This therefore included all mother–infant, sibling–sibling and grandmother–grandoffspring pairs as ‘kin’. We included age and sex of each party as well as kinship as these variables can influence sociosexual and PC affiliation behaviour in bonobos and chimpanzees [15,18,30,40,51]. We included a three-way interaction between species, bystander sex and victim sex in *model 1.1.1*, as well as two-way interactions between species with bystander age, victim age and kinship, respectively, to investigate how bonobos and chimpanzees vary according to how sexual behaviour occurs during triadic contacts. For *model 1.1.2*, we retained the same structure, except species was replaced by group, to test for variation between the two bonobo groups.

In both models, we included the event ID number alongside the identities of all involved parties—aggressor, bystander and victim—in the triadic contact as random effects in a crossed structure. To reduce risk of a type I error, we assessed whether random slope components were theoretically identifiable for all models. Theoretical identifiability was defined as at least three unique values per level of a random effect for covariates and at least two levels with at least two observations per level of a random effect for factors. We included bystander age within aggressor, victim age within bystander and both bystander age and bystander sex within victim. See electronic supplementary material, SI.2 for the structures of *model 1.1.1* and *model 1.1.2*.

For both models, we derived estimates for fixed effects using likelihood-ratio tests (LRTs) by comparing models with null models lacking each fixed effect, respectively, via the drop1 function of the package lme4 in R [67]. If the three-way interaction is found to be non-significant, we remove the three-way term and proceed with a model retaining all two-way interactions to avoid overfitting and aid interpretability.

To directly compare the two species in their overall tendency to use genital contact during triadic victim interactions, we also fitted a reduced model incorporating all fixed effects without interaction terms and retaining the same random effects structure as the full model. We report on this main effect of species in the results and provide the full output for these reduced models in the electronic supplementary material.

We assessed model stability by comparing estimates obtained from the model based on all data with those obtained from models with the levels of the random effects excluded one at a time. We derived confidence intervals using the function bootMer of the package lme4 using 1000 parametric bootstraps and bootstrapping over the random effects too [67]. We *z*-transformed all covariate predictors prior to inclusion in models.

#### 3.1.2. Results

*Model 1.1.1* and *model 1.1.2* each analysed *n* = 784 behaviours that occurred within triadic victim contact interactions during *n =* 194 separate events. Of these contact behaviours, we observed *n* = 322 in bonobos and *n* = 462 in chimpanzees. Across these events, *n =* 81 individuals were observed as uninvolved bystanders (bonobo *n* = 34; chimpanzee *n* = 47), *n* = 60 individuals were observed as victims (bonobo *n* = 28; chimpanzee *n* = 32) and *n* = 42 individuals were observed as aggressors (bonobo *n* = 19; chimpanzee *n* = 22; total *n* includes no aggressor). Across both species, triadic contact interactions featured an average of *M* = 1.88 (s.d. = 1.70, range = 1−12) contact behaviours (bonobos: *M* = 2.39, s.d. = 2.18, range = 1−12; chimpanzees: *M* = 1.62, s.d. = 1.31, range = 1−10). Of these, an average of *M* = 0.50 (s.d. = 0.88, range = 0−8) were categorized as genital contact behaviours (bonobos: *M* = 0.89, s.d. = 1.22, range = 0−8; chimpanzees: *M* = 0.30, s.d. = 0.54, range = 0−3). Frequencies for all coded behaviours across triadic events are reported in electronic supplementary material, SI.3.1.

##### 3.1.2.1. Species-level variation

**Figure 1.**
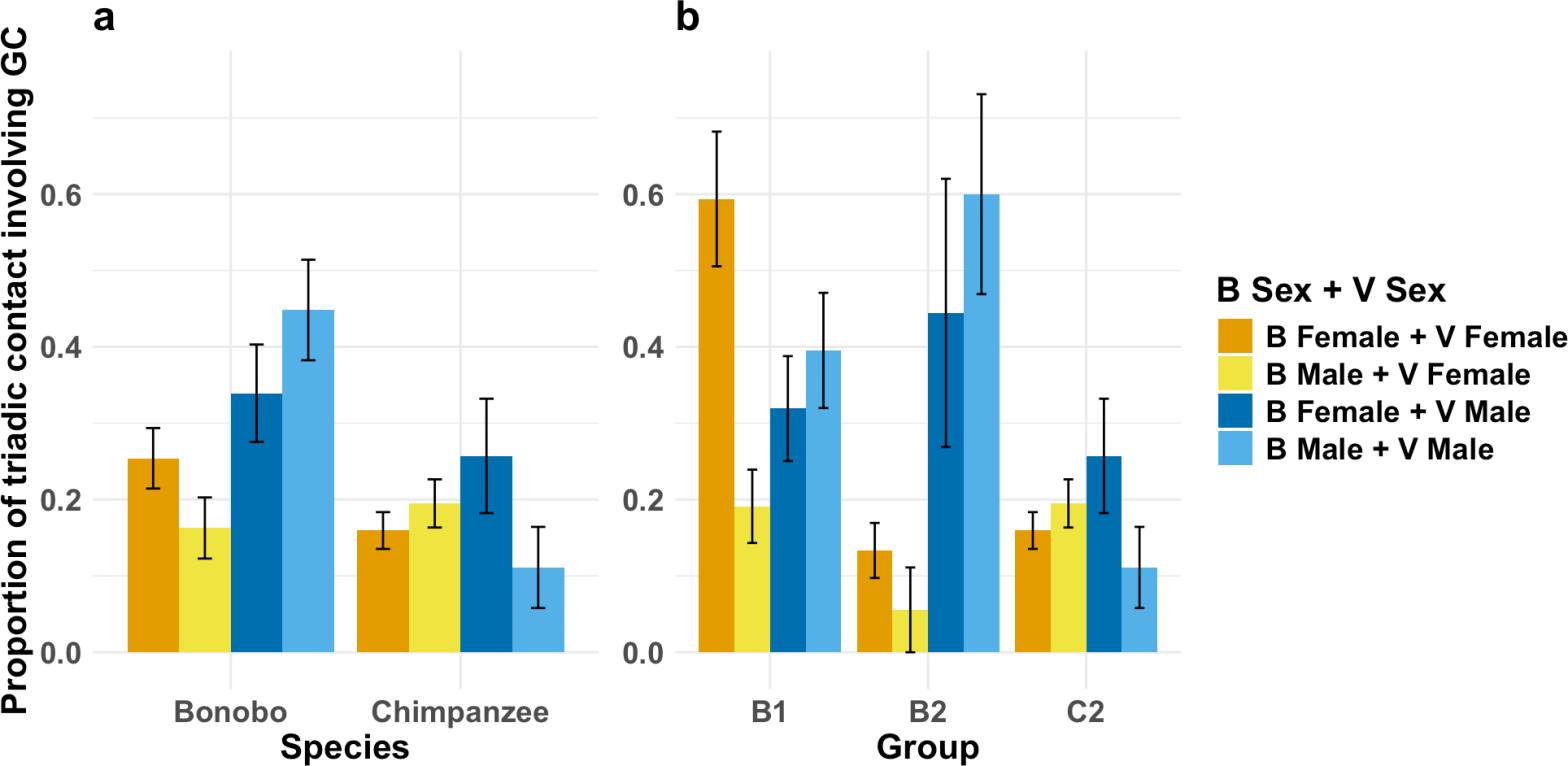
(a) Significant three-way interaction between species, bystander sex and victim sex in whether a triadic contact featured genital contact. Results from *model 1.1.1*. (b) Significant three-way interaction between group, bystander sex and victim sex in use of genital contact during triadic contact. Results from *model 1.1.2*. *Y*-axis shows proportions of triadic contact behaviours that included genital contact. Whiskers show one standard error above and below mean. Abbreviations B = bystander; V = victim; GC = genital contact.

*Model 1.1.1* revealed a significant three-way interaction between species, bystander sex and victim sex on whether a triadic victim contact behaviour involved genital contact (estimate ± s.e. = −3.068 ± 1.489, *χ*^2^ = 4.342, *p* = 0.037). As seen in figure 1a, male–male bonobo bystander–victim triadic contacts were the most likely to feature genital contact, including compared with female–female bonobo pairs. In chimpanzees, male–male triadic contacts were less likely to feature genital contact than both mixed-sex pairings. In addition, non-kin triadic contacts were more likely to involve genital contact than kin pairs, regardless of species (estimate ± s.e. = 2.449 ± 0.467, *χ*^2^ = 36.624, *p* < 0.001). There were no significant main effects for bystander age or victim age, nor were there significant interactions between them and species (see table 2 for estimates and results for respective full-null LRTs).

**Table 2.** Summary of model results for *part 1A*. Species (*model 1.1.1*) and group (*model 1.1.2*) variability in use of genital contact during triadic contacts in *Pan*. Estimates and standard errors, together with confidence intervals, results of likelihood ratio tests and the range of estimates obtained when dropping levels of random effects one at a time. Significant effects at the *p* < 0.05 level are in ***italicised bold***. All fixed and random effect output is provided in electronic supplementary material, SI.4. Abbreviations: B = bystander; V = victim.

part 1A model summary									
term	estimate	s.e.	lower CI	upper CI	χ^2^	z	*p*‐value	min	max
**model 1.1.1 testing species variation in use of genital contacts during triadic victim contact**									
species^a^ × B. age^b^	−0.383	0.412	−1.238	0.433	0.858	−0.930	0.354	−0.659	−0.225
species^a^ × V. age^b^	0.629	0.608	−0.576	2.026	1.099	1.033	0.294	0.306	1.079
species^a^ × kinship^c^	−0.495	0.967	−9.825	1.542	0.264	−0.512	0.607	−1.970	0.796
***species***^***a***^ ***× B. sex***^***d***^ ***× V*. *sex***^***d***^	***−3.068***	***1.489***	***−13.812***	***−0.152***	***4.342***	***−2.060***	***0.037***	***−4.624***	***−2.467***
**model 1.1.2 testing group variation in use of genital contacts during triadic victim contact**									
group^e^ × B. age^b^					0.112		0.946		
group^e^ × V. age^b^					3.167		0.205		
group^e^ × kinship^c^					0.187		0.911		
***group^e^ × B. sex***^d^ ***× V. sex***^d^					***7.088***		***0.029***		

^a^ Reference category = bonobo.

^b^ Z-transformed to a mean of 0 and a s.d. of 1.

^c^ Reference category = kin.

^d^ Reference category = female.

^e^ Reference category = B1

The reduced model revealed a significant main effect of species, whereby bonobo triadic contacts were more likely to involve genital contacts than chimpanzees (estimate ± s.e. = −1.163 ± 0.468, *χ*^2^ = 6.598, *p* = 0.010). Model stability checks revealed all effects for the full and reduced model were robust (see table 2 and electronic supplementary material, SI.4). However, confidence intervals (e.g. three-way interaction: −13.812 to −0.152) indicate uncertainty regarding the magnitude of some effects for the full model. A summary of statistical output for *model 1.1.1* can be found in table 2. The full output for all fixed and random effects of *model 1.1.1* alongside the reduced model is provided in the electronic supplementary material, SI.4.

##### 3.1.2.2. Group-level variation

*Model 1.1.2* revealed a significant three-way interaction between group, bystander sex and victim sex on whether a triadic victim contact behaviour involved genital contact (*χ*^2^ = 7.088, *p* = 0.029). As seen in figure 1b, female–female pairs in B1 show comparable tendencies to use genital contact during triadic contacts to male–male pairs in B2. Triadic contacts between male–male pairs in B2 were more likely to feature genital contact than those between male–male pairs in B1. Furthermore, triadic contacts between female–female pairs in B1 were more likely to feature genital contact than those between female–female pairs in B2. As seen in *model 1.1.1*, there was a significant main effect of kinship (estimate ± s.e. = 2.640 ± 0.906, *χ*^2^ = 2.915, *p* < 0.001). There were no significant interaction effects between group and bystander age, group and victim age or group and kin (see table 2). However, as in *model 1.1.1*, confidence intervals indicate substantial uncertainty regarding the magnitude of some effects for the full model and stability checks indicated that some effects for the full model were not robust (see table 2 for a summary of output from *model 1.1.2*). Thus, results from this model should be interpreted with caution. The full output for all fixed and random effects of *model 1.1.2* is provided in the electronic supplementary material, SI.5.

### 3.2. Part 1B: Reconciliation

#### 3.2.1. Analysis

To test if bonobos are more likely than chimpanzees to use genital contact during reconciliatory interactions, we fitted a GLMM in RStudio (v. 1.3.1093 [66]) using the function glmer of the package lme4 (v. 1.1-28 [67]). Specifically, we analysed a behaviour-level data frame of *n* = 77 observation rows, where one row constituted one contact behaviour that occurred during *n* = 44 PC events that featured one or more reconciliatory contact between a previous aggressor–victim pair. We fitted one GLMM with a binomial error distribution to test general species differences in tendencies to use genital contacts during reconciliation (*model 1.2*). The response variable was a dichotomous 1/0 variable (1 = behaviour involved genital contact; 0 = behaviour did not involve genital contact).

For *model 1.2*, the fixed-effects structure included species, aggressor age, aggressor sex, victim age and victim sex of the aggressor–victim pairing. We did not include kin as kin conflict rarely occurred and we only observed *n* = 1 instance of a reconciliatory contact between a kin pair. Inclusion of interactions in this model was not possible due to this reduced data frame. We included the event ID number alongside the identities of the aggressor and victim as random effects in a crossed structure. No random slopes were theoretically identifiable. See electronic supplementary material, SI.6 for the structure of *model 1.2*.

We derived estimates for fixed effects using LRTs by comparing full models with null models lacking each fixed effect respectively via the drop1 function of the package lme4 in R [67]. We assessed model stability and derived confidence intervals using the same methods as in *part 1A* [67] and likewise *z*-transformed all covariate predictors prior to inclusion in models.

#### 3.2.2. Results

*Model 1.2* analysed *n* = 77 behaviours that occurred within reconciliatory contact interactions during *n =* 44 separate events. Of these contact behaviours, we observed *n* = 28 in bonobos and *n* = 49 in chimpanzees. Across these events, *n* = 29 individuals were observed as victims (bonobo *n* = 10; chimpanzee *n* = 19), and *n* = 16 individuals were observed as aggressors (bonobo *n* = 7; chimpanzee *n* = 9). Across both species, reconciliatory contact interactions featured an average of *M* = 1.75 (s.d. = 1.33, range = 1−6) contact behaviours (bonobos: *M* = 2.15, s.d. = 1.68, range = 1−6; chimpanzees: *M* = 1.58, s.d. = 1.15, range = 1−6). Of these, an average of *M* = 0.43 (s.d. = 0.62, range = 0−2) were categorized as genital contact behaviours (bonobos: *M* = 0.92, s.d. = 0.64, range = 0−2; chimpanzees: *M* = 0.23, s.d. = 0.50, range = 0−2). Frequencies for all coded behaviours across reconciliatory events are reported in electronic supplementary material, SI.3.2.

##### 3.2.2.1. Species-level variation

**Figure 2.**
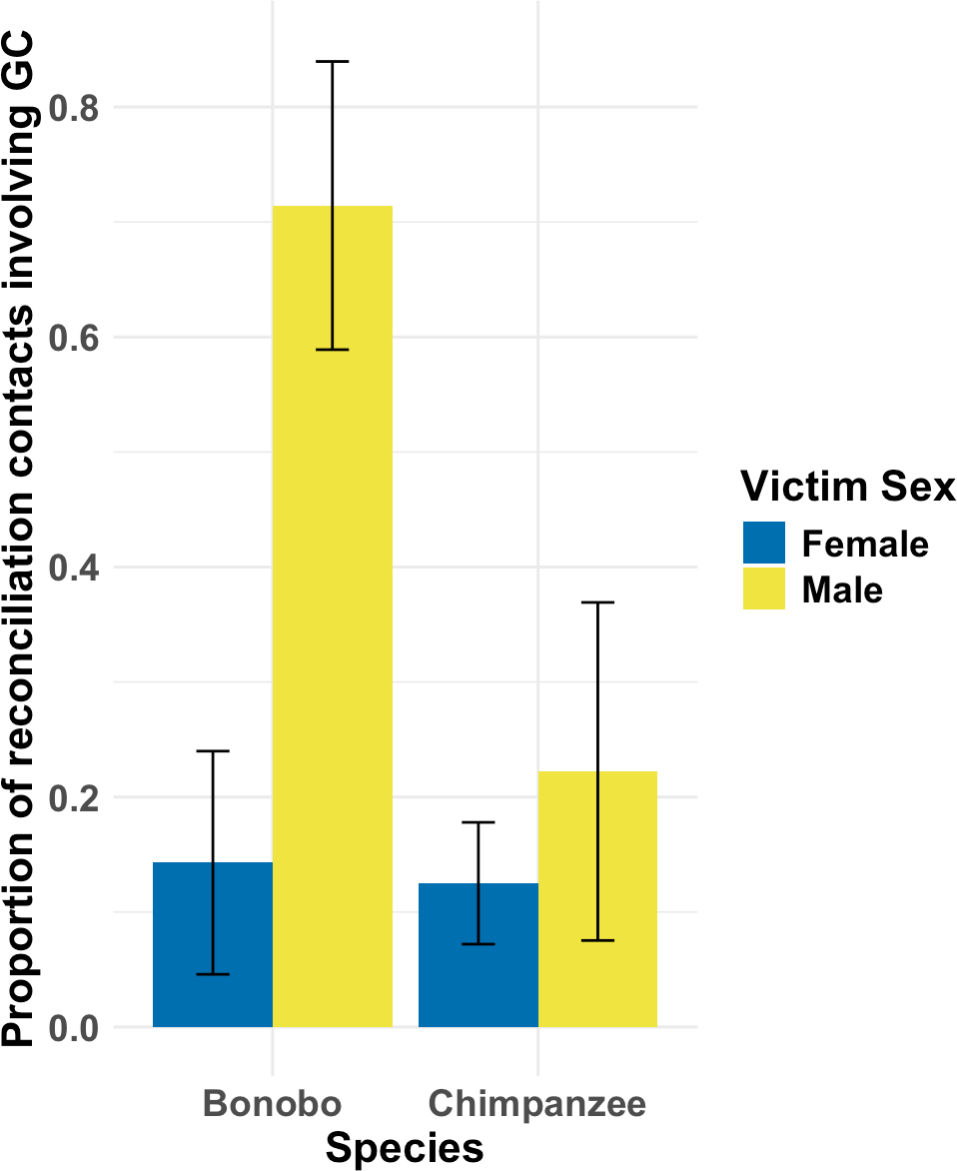
Significant main effect of victim sex regarding use of genital contact during reconciliation contacts. *Y*-axis shows proportions of reconciliation contact behaviours that included genital contact. Results from *model 1.2.* Whiskers show one standard error above and below mean. Abbreviation GC = genital contact.

*Model 1.2* revealed a significant effect of species, whereby bonobo reconciliatory contacts were more likely to involve genital contacts than chimpanzees (estimate ± s.e. = −2.655 ± 1.690, *χ*^2^ = 4.123, *p* = 0.042). In addition, reconciliatory contacts with male victims were more likely to involve genital contact than those involving female victims (estimate ± s.e. = 2.332 ± 1.082, *χ*^2^ = 6.314, *p* = 0.012). However, figure 2 indicates this trend may have been driven by data from bonobos. While model stability checks revealed these effects may be robust across individuals and events (see table 3), confidence intervals revealed substantial uncertainty regarding the magnitude of these effects. For these reasons, and due to paucity of data from both species, we recommend interpreting all results relating to *model 1.2* with caution. There were no significant effects for aggressor age, aggressor sex or victim age (see table 3 for full statistical output for *model 1.2*).

**Table 3.** Full results for *model 1.2* testing species differences in use of genital contact during reconciliation contacts in *Pan*. Estimates and standard errors, together with confidence intervals, results of likelihood ratio tests and the range of estimates obtained when dropping levels of random effects one at a time. Significant effects at the *p* < 0.05 level are in ***italicised bold***.

model 1.2: main effects model testing species differences in use of genital contact during reconciliatory contacts									
term	estimate	s.e.	lower CI	upper CI	χ^2^	z	*p*‐value	min	max
*fixed effects*									
(intercept)	−0.459	0.927	−4.266	1.821		−0.495	0.621	−1.032	0.384
***species^a^***	***−2.655***	***1.690***	***−19.918***	***−0.020***	***4.123***	***−1.570***	***0.042***	***−4.976***	***−1.969***
aggressor sex^b^	0.002	0.958	−2.385	14.611	0.000	0.002	0.998	−0.985	1.145
***victim sex^b^***	***2.332***	***1.082***	***0.315***	***12.023***	***6.314***	***2.155***	***0.012***	***1.164***	***3.394***
aggressor age^c^	0.304	0.564	−1.455	2.058	0.281	0.539	0.596	−0.021	0.678
victim age^c^	0.618	0.595	−0.928	3.316	1.161	1.039	0.281	0.247	1.030
*random effects*									
aggressor ID	0.691							0.000	1.485
event ID	0.314							0.000	0.873
victim ID	0.000							0.000	0.000

^a^ Reference category = bonobo.

^b^ Reference category = female.

^c^ *Z*-transformed to a mean of 0 and a s.d. of 1.

## 4. Part 2: Variability in *Pan* pre-feeding genital contact

### 4.1. Analysis

For these analyses, we used the same approach as *part 1*, fitting frequentist GLMMs in RStudio (v. 1.3.1093 [66]) using the function glmer of the package lme4 (v. 1.1-28 [67]). To compare bonobos’ and chimpanzees’ tendencies to use genital contacts during a pre-feeding period, we analysed a behaviour-level data frame of *n* = 1670 observation rows, where one row constituted one contact behaviour that occurred during *n* = 45 pre-feeding periods (bonobo pre-feeding event *n* = 28; chimpanzee pre-feeding event *n* = 17). The response variable was a dichotomous 1/0 variable (1 = behaviour involved genital contact; 0 = behaviour did not involve genital contact).

As in *part 1A*, we fitted two binomial GLMMs (*model 2.1* and *model 2.2*) to investigate species and group differences in the use of genital contact during pre-feeding contacts. For *model 2.1*, the fixed-effects structure included species, initiator age, initiator sex, recipient age, recipient sex and kinship of the initiator–recipient pairing. We included a three-way interaction between species, bystander sex and victim sex in *model 2.1*, as well as two-way interactions between species with initiator age, recipient age and kinship, respectively, to investigate how bonobos and chimpanzees vary according to how sexual behaviour occurs during pre-feeding contacts. For *model 2.2*, we retained the same structure, except species was replaced by group, to test for variation between the *Pan* groups. For *model 2.2*, we applied a reduced data frame lacking group B3 due to no observations of male–male pairs interacting. We also proceeded without an interaction between group and kinship, which led to destabilized model estimates.

In both models, we included the session number alongside the identities of the initiator and recipient as random effects in a crossed structure. For *model 2.1*, we also included group as a random intercept and the following theoretically identifiable random slopes: recipient sex within initiator, initiator sex within recipient and initiator age, initiator sex, kinship and species within session number. For *model 2.2*, we included the following theoretically identifiable random slopes: recipient age within initiator ID, initiator age and initiator sex within recipient ID and initiator sex and kinship within session number. All factor variables were dummy-coded and centred prior to their inclusion as random slopes. See electronic supplementary material, SI.7 for the structure of *model 2.1* and *model 2.2*.

We derived estimates for fixed effects, model stability and confidence intervals using the same methods as *part 1* [67] and likewise *z*-transformed all covariate predictors prior to inclusion in models. As in *part 1A*, if the three-way interaction is found to be non-significant, we remove the three-way term and proceed with a model retaining all two-way interactions to avoid overfitting and aid interpretability. In addition, to directly compare the two species in their overall tendency to use genital contact during pre-feeding interactions, we also fitted a reduced model incorporating solely main fixed effects.

### 4.2. Results

*Model 2.1* analysed *n* = 1670 behaviours that occurred within pre-feeding affiliative contact interactions during *n =* 45 separate pre-feeding events. Of these contact behaviours, we observed *n* = 508 in bonobos and *n* = 1162 in chimpanzees. Across these events, *n =* 107 individuals initiated one or more contact behaviours (bonobo *n* = 43; chimpanzee *n* = 64) and *n* = 112 individuals received one or more contact behaviours (bonobo *n* = 45; chimpanzee *n* = 67). Across both species, pre-feeding dyadic interactions featured an average of *M* = 1.85 (s.d. = 1.56, range = 1−13) contact behaviours (bonobos: *M* = 2.09, s.d. = 1.91, range = 1−12; chimpanzees: *M* = 1.77, s.d. = 1.40, range = 1−13). Of these, an average of *M* = 0.35 (s.d. = 0.66, range = 0−5) were categorized as genital contact behaviours (bonobos: *M* = 0.46, s.d. = 0.90, range = 0−5; chimpanzees: *M* = 0.30, s.d. = 0.54, range = 0−4). *Model 2.2* analysed *n* = 1565 behaviours that occurred within pre-feeding affiliative contact interactions during *n =* 35 separate pre-feeding events for groups B1, B2, C1 and C2. Frequencies for all coded behaviours across pre-feeding sessions are reported in electronic supplementary material, SI.3.3.

#### 4.2.1. Species-level variation

**Figure 3.**
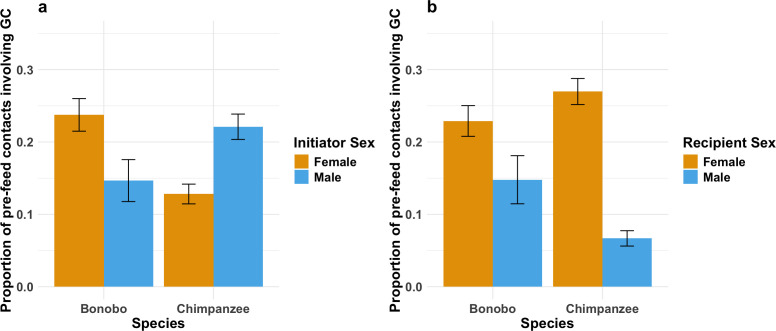
(a) Significant interaction effect between species and initiator sex and (b) significant main effect of recipient sex regarding use of genital contact during pre-feed contacts. *Y*-axis shows proportions of pre-feed behaviours that included genital contact. Whiskers show one standard error above and below mean. Abbreviation GC = genital contact.

*Model 2.1* initially revealed a non-significant three-way interaction between species, initiator sex and recipient sex (*χ*^2^ = 3.656, *p* = 0.056; see results for full-null LRTs in electronic supplementary material, SI.8). Thus, we removed this three-way term and proceeded with only two-way terms. The reduced model demonstrated a significant interaction between species and initiator sex (estimate ± s.e. = 1.354 ± 0.618, z = 2.192, *p* = 0.027), whereby female bonobos and male chimpanzees were each more likely to use behaviours involving genital contacts during pre-feeding interactions than their respective counterparts (i.e. male bonobos and female chimpanzees; see figure 3). Furthermore, regardless of species, contacts received by females broadly tended to involve genital contact more than those received by males (estimate ± s.e. = −1.244 ± 0.529, *z* = −2.353, *p* = 0.019; see figure 3). In addition, while kinship did not interact with species, non-kin pre-feeding contacts were significantly more likely to involve genital contact than those between kin pairs (estimate ± s.e. = 2.264 ± 0.623, *z* = 3.631, *p* < 0.001). There were no significant interactions or main effects involving initiator age or recipient age (see table 4 for estimates and results of respective full-null LRTs and electronic supplementary material, SI.9 for random effect estimates).

**Table 4.** Results for reduced *model 2.1* testing species variability in use of genital contact during pre-feeding contacts in *Pan*. Estimates and standard errors, together with confidence intervals, results of likelihood ratio tests and the range of estimates obtained when dropping levels of random effects one at a time. Significant effects at the *p* < 0.05 level are in ***italicised bold***. All fixed and random effect output is provided in electronic supplementary material, SI.9. Abbreviations: I = Initiator; R = Recipient.

model 2.1: reduced model testing species variation in use of genital contact during pre-feeding affiliative contacts (only two-way interactions included)									
term	estimate	s.e.	lower CI	upper CI	χ^2^	z	*p*‐value	min	max
*fixed effects*									
***(intercept)***	***−2.970***	***0.700***	***−5.044***	***−1.786***		***−4.271***		***−4.429***	***−2.337***
species^a^	0.319	0.802	−1.127	2.431		0.398	(—)	−0.338	1.774
I. sex^b^	−0.867	0.519	−1.971	0.052		−1.672	(—)	−1.536	−0.573
***R. sex^b^***	***−1.244***	***0.529***	***−2.442***	***−0.279***		***−2.353***	***0.019***	***−2.177***	***0.370***
***species***^***a***^ ***×*** ***I. sex***^***b***^	***1.354***	***0.618***	***0.163***	***2.638***	***4.876***	***2.192***	***0.027***	***1.054***	***2.192***
species^a^ × R. sex^b^	−1.108	0.638	−2.275	0.286	2.816	−1.736	0.093	−2.637	0.077
I. sex^b^ × R. sex^b^	0.357	0.478	−0.635	1.332	0.556	0.746	0.456	−0.102	0.713
I. age^c^	0.572	0.316	−0.088	1.239		1.809	0.070	0.313	0.788
species^a^ × I. age^c^	−0.289	0.361	−1.040	0.455	0.630	−0.802	0.427	−0.551	−0.030
R. age^c^	0.005	0.341	−0.651	0.596		0.016	0.987	−2.157	0.330
species^a^ × R. age^c^	0.211	0.383	−0.490	0.993	0.310	0.551	0.578	−0.131	2.346
***kinship^d^***	***2.264***	***0.623***	***1.239***	***4.287***		***3.631***	***<0.001***	***1.356***	***2.994***
species^a^ × kinship^d^	−0.773	0.725	−2.941	0.418	1.175	−1.067	0.278	−1.491	0.209

^a^ Reference category = bonobo.

^b^ Reference category = female.

^c^ *Z*-transformed to a mean of 0 and a s.d. of 1.

^d^ Reference category = kin.

The main effects model revealed no significant main effect of species, whereby bonobos and chimpanzees did not differ in their tendency for pre-feed contacts to feature genital contact (estimate ± s.e. = −0.146 ± 0.392, *χ*^2^ = 0.138, *p* = 0.710). Model stability checks revealed all effects for the reduced and main effects models were robust (see table 4 and electronic supplementary material, SI.9–10). Statistical output for *model 2.1* can be found in table 4 and output for the main effects model can be seen in electronic supplementary material, SI.10.

#### 4.2.2. Group-level variation

**Figure 4.**
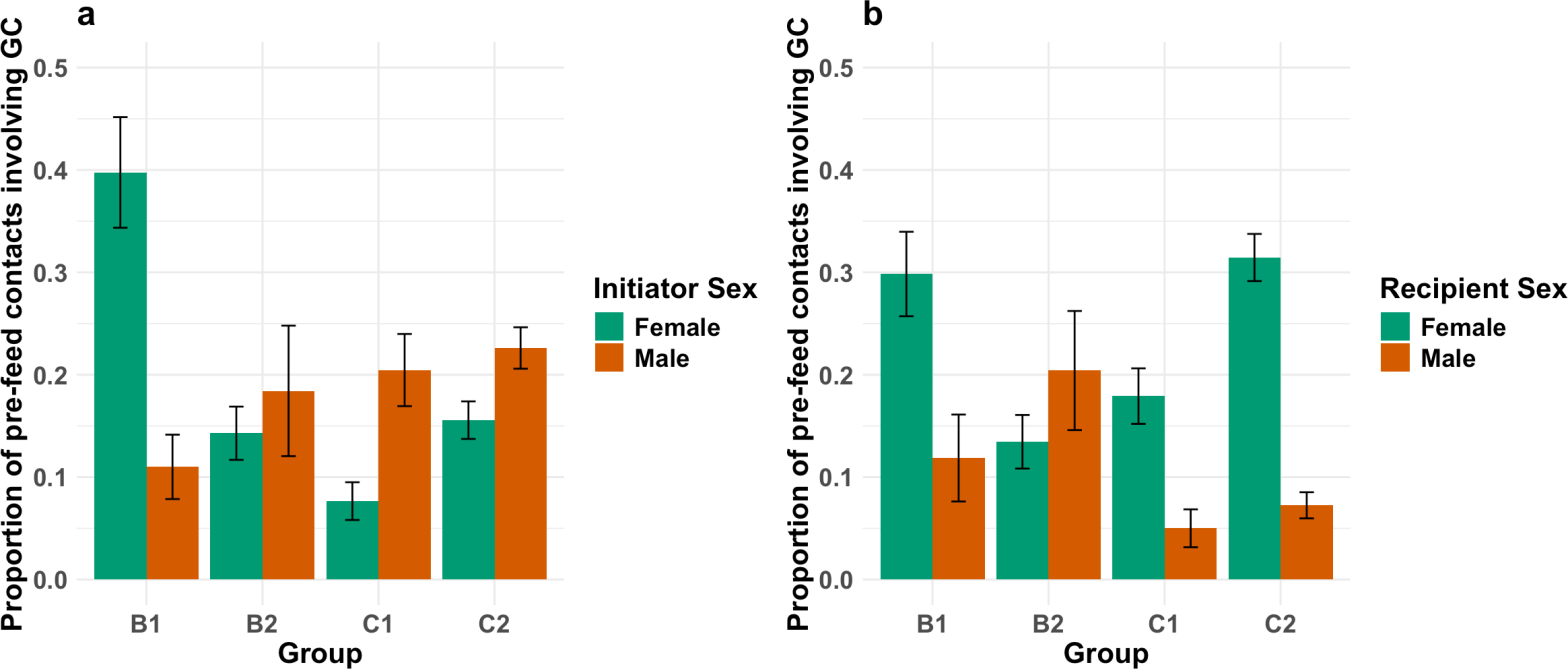
(a) Significant interaction effect between group and initiator sex and (b) significant interaction effect between group and recipient sex regarding use of genital contact during pre-feed contacts. *Y*-axis shows proportions of pre-feed behaviours that included genital contact. Whiskers show one standard error above and below mean. Abbreviation GC = genital contact.

The full model revealed no significant three-way interaction between group, initiator sex and recipient sex (*χ*^2^ = 6.086, *p* = 0.107; see results for full-null LRTs in electronic supplementary material, SI.10). Thus, we removed this three-way term and proceeded with only two-way terms. We found a significant interaction between group and initiator sex (*χ*^2^ = 11.116, *p* = 0.011; see figure 4) and between group and recipient sex (*χ*^2^ = 13.942, *p* = 0.003; see figure 4). In both cases, contacts involving females in group B1 were more likely to include genital contact than males in B1 and both sexes in B2. In B2, the two sexes did not appear to vary regarding which sex initiates or receives genital contact. In the chimpanzee groups, males initiated more genital contact behaviours than females, with higher rates for female initiation in C2 than C1. Conversely, females were more likely to receive genital contact behaviours than males in both C1 and C2, but females were more likely to receive in C2 than C1.

*Model 2.2* also revealed a significant main effect for kinship (estimate ± s.e. = 1.748 ± 0.426, *χ*^2^ = 15.795, *p* < 0.001). We also found a significant interaction between group and recipient age (*χ*^2^ = 15.055, *p* = 0.002). Pre-feed contacts featuring genital contact in group B1 tended to be initiated by younger individuals more than older individuals, compared with other groups where there were no clear trends (see electronic supplementary material, SI.13). However, the predicted probabilities for this interaction feature a considerable range of uncertainty, indicating that this result should be interpreted with caution. Model stability checks revealed these effects were robust (see table 5). Statistical output for the reduced *model 2.2* can be found in table 5.

**Table 5.** Results for reduced *model 2.2* testing group variability in use of genital contact during pre-feeding contacts in *Pan*. Estimates and standard errors, together with confidence intervals, results of likelihood ratio tests and the range of estimates obtained when dropping levels of random effects one at a time. Significant effects at the *p* < 0.05 level are in ***italicised bold***. All fixed and random effect output is provided in electronic supplementary material, SI.12. Abbreviations: I = Initiator; R = Recipient.

model 2.2: reduced model testing group variation in use of genital contact during pre-feeding affiliative contacts (only two-way interactions included)									
term	estimate	s.e.	lower CI	upper CI	χ^2^	z	*p*‐value	min	max
*fixed effects*									
(intercept)	−1.180	0.681	−2.517	0.123		−1.733	0.083	−1.655	−0.373
group [B2]^a^	−5.655	1.854	−10.974	−2.663		−3.051	(—)	−6.917	−4.373
[C1]^a^	−2.062	0.732	−3.611	−0.659		−2.818	(—)	−2.794	−1.555
[C2]^a^	−1.488	0.633	−2.698	−0.303		−2.351	(—)	−2.251	−0.996
I. sex^b^	−2.558	0.919	−4.885	−1.000		−2.785	(—)	−3.209	−1.883
R. sex^b^	−1.502	0.781	−3.900	−0.102		−1.924	(—)	−2.604	−0.745
***group^a^ x I. sex^b^***					***11.116***		***0.011***		
***group^a^ x R. sex^b^***					***13.961***		***0.003***		
I. sex^b^ x R. sex^b^	0.165	0.473	−0.777	1.056	0.122	0.350	0.727	−0.089	0.393
I. age^c^	−0.358	0.534	−1.533	0.594		−0.671		−1.014	0.054
group^a^ x I. age^c^					1.542		0.673		
R. age^c^	0.943	0.467	−0.028	1.957		2.020		0.528	1.437
***group^a^ x R. age^c^***					***15.111***		***0.002***		
***kinship^d^***	***1.748***	***0.426***	***1.094***	***2.566***	***15.795***	***4.100***	***<0.001***	***1.553***	***2.039***

^a^ Reference category = B1.

^b^ Reference category = female.

^c^ *Z*-transformed to a mean of 0 and a s.d. of 1.

^d^ Reference category = kin.

## 5. Discussion

Combining naturalistic observations and a controlled feeding experiment, we conducted a first direct comparison of genital contact behaviour in our two closest cousins, the bonobos and chimpanzees. Although we found some species differences, we demonstrated considerable overlap in their use of genital contacts during the following three socially tense contexts: (i) PC triadic victim interactions, (ii) PC reconciliation, and (iii) pre-feeding competition. As predicted, bonobos were more likely to use genital contacts during PC triadic contacts and reconciliation. However, contrary to predictions, the rate of genital contacts was comparable between the species during pre-feeding competition. Furthermore, during pre-feeding competition, female bonobos and male chimpanzees initiated genital contacts at comparable levels and both more so than male bonobos and female chimpanzees. However, female members of both species were more likely to receive genital contacts than males, contrary to our prediction that male chimpanzees would both initiate and receive the most genital contacts.

In sum, although bonobos have a reputation as being hyper-sexual [68,69], we demonstrate here considerable overlap between bonobos and chimpanzees in their use of genital contact behaviour to navigate social tension [19,30]. While we were unable to test direct functions with our data, all of the affiliative contexts we investigated—PC triadic contacts, reconciliation and pre-feeding affiliation—are associated with stress reduction, restoration of social bonds and prevention of conflict escalation broadly [40,42,49,60]. By focusing on these contexts, we show evidence that both species use genital contacts at comparable tendencies during periods where risk of aggression and social instability is elevated.

Sociosexual behaviour can be used as a form of social tension and stress-buffering in humans, bonobos and chimpanzees [1,2,5,6,18,27,30,48]. Thus, in line with previous findings, the overlap we show supports the notion that the use of sexual contacts may have functioned adaptively to manage social bonds and mitigate conflict in our shared evolutionary ancestry with the *Pan* apes. In addition, our findings highlight the importance of considering group-level variation in the expression of sexual behaviour, as such variation may offer insights into the complex socio-emotional landscapes within and between groups during socially tense contexts [70]. Overall, our study highlights the need to focus on the flexible, context-dependent deployment of sexual behaviours in both species, as well as others known to perform sexual behaviours in social settings.

Both *Pan* apes performed a diverse repertoire of affiliative contact behaviours thought to have reassuring functions in specific contexts, such as engaging in friendly contact with distressed victims after fights [40,60] or prior to feeding, a context of potential competition risk. This repertoire encompasses a rich subset of sexual contacts including genital touching, GG contacts and mounting (with and without intromission) that occur in various contexts [12,18,30,43]. Sociosexual behaviour appears to be particularly pronounced and habitual in bonobos compared with other primates, including chimpanzees [11,13], where it is thought to be linked with cooperation, feeding tolerance, food sharing [20,22] and potential avoidance of aggression [71]. However, genital contacts between individuals of all age and sex combinations can occur in both *Pan* species [19,30], something our results show.

While tendencies for sexual forms of PC affiliation were higher in bonobos, chimpanzees still used genital contacts in this context, indicating a species overlap [18]. This parallels previous studies of chimpanzee consolation and other PC interactions, where genital touching and mounting are often reported (e.g. [34,50,72]). Sexual behaviour may function to alleviate social tension and reduce stress in bonobos [12,18]. Our results support this view and indicate that genital contacts may share a similar function in chimpanzees [30].

Chimpanzees appear to have a wider overall repertoire of reassurance behaviours than bonobos, as they are known to engage in several vulnerable mouth-to-body behaviours—including body kissing and finger/hand in mouth [34,72–74]—that have not been reported in bonobos. Regardless, genital contacts still constituted a sizable portion of chimpanzee triadic, reconciliatory and pre-feeding contact behaviour. Engaging in vulnerable behaviour in risky contexts is thought to promote trust between possible competitors and test social bonds [75–77]. The methods employed in this study mean it would be difficult to ascertain stress-reducing or social bond testing functions in these cases, particularly in the pre-feeding context, as we recorded affiliative contacts for every dyadic interaction in large populations. However, both mouth-to-body and genital contact behaviour place one at comparable risk during a period where the risk of aggression is elevated, especially for chimpanzees [78–80].

Thus, genital contacts may constitute the dominant form of reassurance in bonobos [10,12], whereas in chimpanzees they may represent one of several possible forms [30,33,35,43,44]. Our findings indicate that the tendency to use genital contacts during social tension may instead depend on context in chimpanzees. For example, conflict appears to be riskier in chimpanzees than in bonobos [78–80], and during PC periods, redirected or renewed aggression can be common [50,72]. Other affiliative contacts—such as touching, embracing or mouth-to-body behaviours—may be preferred during PC contexts in chimpanzees to communicate benign intentions more clearly. In contrast, during a pre-feeding competitive context, while tension may be high, individuals may have more trust to initiate genital contacts if conflict has not yet occurred. Deciphering communicative intentions in great apes is difficult, yet a direct investigation into comparing forms and outcomes of consolation behaviours could reveal whether use of a specific contact is dependent on variables beyond the demography of the parties.

In line with previous findings, genital contacts were offered by and received by individuals of all ages and sexes in both species. Age did not predict the use of genital contact during triadic or reconciliation interactions in bonobos or in chimpanzees. During the pre-feeding period, older apes were more likely to initiate genital contacts than younger apes, which could indicate that using sexual behaviour in reassurance contexts is a learned behaviour. In addition, while some maternal kin interactions involved genital contacts, non-kin behaviours were much more likely to involve genital contact between non-kin pairs. It may be that genital contacts are less common among kin to avoid incest and particularly common between non-kin close social partners to prevent conflict, reinforce social bonds and foster closeness during feeding, akin to Moscovice *et al*. [22] for bonobos. A follow-up could investigate whether the genital contact types vary between different maternal sex pairings (i.e. female–male or same-sex kin-related pairs). Demuru *et al*. [81] found that females who were less socially bonded were more likely to synchronize regarding their maximum sexual swelling. As synchronization is also associated with increased GG rubbing in bonobos [81], our findings support the notion that sexual contacts may be used to strengthen social bonds in certain contexts.

As previously proposed [22], the use of sociosexual behaviour during pre-feeding periods may function to foster greater social closeness during feeding for both species. Due to limited data and model overcomplexity, it was not possible to assess how dyadic social relationships affect the tendency to use genital contacts in these tense contexts. However, in this study, genital contacts were performed across groups, ages and sexes of sanctuary-living bonobos and chimpanzees [19,30]. Future research could directly target assessing this function. Collecting more observations and directly assessing grooming and play relationship association networks could reveal the importance of sex for fortifying alliances beyond kin relationships during competitive or tense periods that may threaten group cohesiveness and stability [82]. In addition, studying the directionality and form of sexual encounters may reveal group-specific trends that parallel with their respective social climates. Finally, while difficult to study directly in our data due to the presence of contraceptives, investigating the influence of female sexual swellings on the forms and prevalence of genital contact behaviour in these contacts would further elucidate the diversity and flexibility of the *Pan* sexual repertoires.

Overall, these findings support the notion that beyond a purely reproductive function, our closest living relatives possess rich sociosexual lives, whereby affiliative genital contacts appear to contribute to the management of social relationships and periods of social tension [2,12,30]. The reputation that bonobos have for being the species more focused on sociosexual interactions is only partly supported by these findings given the occurrence of genital contacts in both species in comparable contexts. In fact, the importance of sexuality in chimpanzee social life generally should not be understated, particularly in relation to social tension management. However, bonobos appear to use sociosexual interactions more habitually in other contexts than chimpanzees, including those involving social tension. Continued comparative research into between- and within-species trends can shed greater light on the overall significance of sexual behaviour for regulating social relationships in Hominid evolutionary history.

## Data Availability

All code and data are accessible via an OSF folder at the following link: [57]. Supplementary material is available online [83].
